# Acute Myocardial Infarction as an Initial Presentation of Protein C and Protein S Deficiency Followed by Dilated Cardiomyopathy in a Young Male

**DOI:** 10.7759/cureus.4492

**Published:** 2019-04-17

**Authors:** Faryal Tahir, Zainab Majid, Bushra Majid, Sherbano Khan

**Affiliations:** 1 Internal Medicine, Dow University of Health Sciences, Karachi, PAK; 2 Surgery, Dow University of Health Sciences, Karachi, PAK

**Keywords:** myocardial infarction, thrombophilia, arterial thrombosis, protein c deficiency, protein s deficiency, dilated cardiomyopathy, ejection fraction, ventricular systolic function, pulmonary edema

## Abstract

Protein C and protein S are vitamin K dependent anti-coagulant proteins required for the inhibition of activated protein V and VIII. In an inherited thrombophilia, hypercoagulability caused by the deficiency of protein C and protein S predisposes an individual to increased risk of thromboembolism (TE) that could herald as a venous thromboemboilsm (VTE) in the leg, pulmonary embolism (PE), stroke, or Budd-Chiari syndrome. However, very rarely does inherited thrombophilia cause coronary artery thrombosis leading to the development of myocardial infarction (MI). We report a case of a young male with combined protein C and protein S deficiency who presented with acute MI, worsened ventricular systolic function, and progressive declination of ejection fraction (EF) secondary to dilated cardiomyopathy (DCM).

## Introduction

Cardiovascular disorders (CVD) remain the leading cause of mortality and morbidity globally, and the incidence continues to increase. Myocardial infarction (MI), the commonest form of CVD, mostly occurs due to an atherosclerotic plaque and manifests usually in adults [1]. An earlier onset of CVD should alarm the healthcare professionals as an inherited disorder could be the likely culprit. A state of hypercoagulability caused by the deficiency of protein C and protein S predisposes an individual to an increased risk of thromboembolism (TE) that could herald as a venous thromboembolism (VTE) in the leg, pulmonary embolism (PE), stroke, or Budd-Chiari syndrome [2]. While protein C and protein S deficiency have been reported in disorders like nephrotic syndrome, inflammatory bowel disease (IBD), human immunodeficiency virus (HIV), varicella, multiple myeloma, sepsis, etc., an inherited deficiency of these proteins is not an oft-sighted entity [3]. Protein C and protein S are vitamin K dependent anti-coagulant proteins where the former requires the latter for its activation and inhibition of activated protein V and VIII [4]. The overall incidence of venous thrombotic events in the setting of protein C and/or protein S deficiency is reported to be much higher than that of arterial thrombosis, with a ratio of almost 24 to 1 [5]. However, very rarely does inherited thrombophilia cause coronary artery thrombosis leading to the development of MI. Other associated factors like fibrinogen, heparin cofactor I, lipoprotein (a), and anticardiolipin antibodies may also be implicated in the formation of arterial thrombosis [4]. Nevertheless, the incidence is rare and no such study in Pakistan has demonstrated its presence. Hence, this may be under reported. Here, we present this case of a 27-year-old male with a past history of MI due to protein C and protein S deficiency, who presented to Civil Hospital, Karachi (CHK) with pulmonary edema secondary to dilated cardiomyopathy (DCM).

## Case presentation

**Figure 1:**
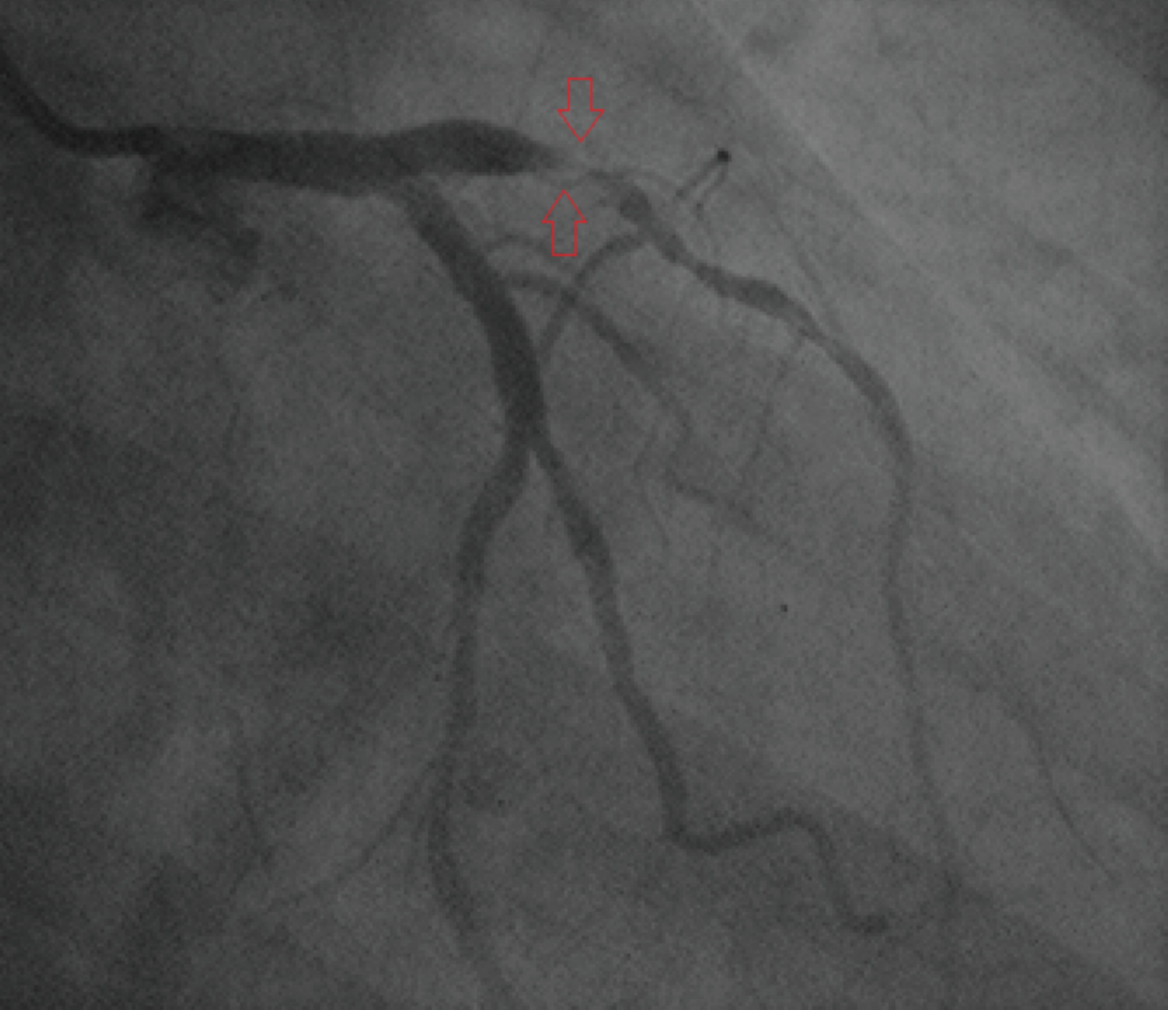
Angiogram showing thrombotic occlusion of the proximal LAD artery. LAD, left anterior descending.

Our patient, a 27-year-old male with a significant medical history of tobacco use and a positive family history of CVD (father had fatal MI at 38 years of age), had presented in 2016 with shortness of breath (SOB) and acute retrosternal chest pain. His electrocardiography (ECG) showed ST-elevation myocardial infarction (STEMI) with ST elevations in leads V1-V4 and ST depressions in the reciprocal leads. Echocardiogram (echo) showed severe left ventricular dysfunction (LVD), akinetic left ventricular (LV) apex, and an ejection fraction (EF) of 25%. Cardiac catheterization revealed thrombotic occlusion of the left anterior descending (LAD) artery (Figure 1) in which percutaneous coronary intervention (PCI) with stent placement was performed as part of immediate management for anterior wall myocardial infarction (AWMI). Deficiency of protein C and protein S was diagnosed through coagulation profile; values seen were 35% and 56%, respectively, as shown in Table 1.

**Table 1:** Coagulation profile PT, prothrombin time; APTT, activated partial thromboplastin time; INR, international normalized ratio; IgG, immunoglobulin G; IgM, immunoglobulin M; ANA, antinuclear antibody.

Test name	Patient's value	Reference range
PT test	11.4 s	11-15 s
APTT test	17.6 s	26-36 s
INR	1.09	1
Protein C	35%	70%-140%
Protein S	56%	70%-123%
Factor V Leiden mutation	Negative	-
Anti cardiolipin antibody IgG	Nonreactive	-
Anti cardiolipin antibody IgM	Nonreactive	-
ANA test	Nonreactive	-
Serum homocysteine	14.67 umol/L	5.46-16.4 umol/L

Lipid profile was found to be normal. After adequate management, he was discharged on aspirin, clopidogrel, rosuvastatin, and loop diuretic. After one year our patient, found to be noncompliant to medications, was admitted again with the complaint of SOB, fever, and generalized edema for which he was managed as a case of ischemic heart disease (IHD). Echo then showed declining EF of 20% with prominent DCM. Computed tomography (CT) scan of the chest and abdomen revealed loculated pleural effusion, dilated pulmonary vessels, and gross ascites in the presence of cor pulmonale.

Now, the patient arrived in emergency room (ER) on February 28th, 2019 with severe dyspnea, pedal edema, cellulitis of left leg up to the knee, and fever. Pedal edema was bilateral without periorbital swelling whereas the SOB of New York Heart Association (NYHA) class III along with orthopnea was accompanied with white productive cough. A complaint of bilateral blurred vision for the past one week was also noted.

Physical examination showed an anemic, jaundiced and mildly icteric young male, with a blood pressure (BP) of 110/80 mmHg, respiratory rate (RR) of 22 breaths/min, temperature of 100 degree Farenheit with raised jugular venous pressure (JVP) and engorged veins. Fine rising crepitations were auscultated in lower and mid zones of chest with a pansystolic murmur all over the precordium. Abdomen was mildly tender with palpable liver, whereas the central nervous system (CNS) was grossly intact.

**Figure 2:**
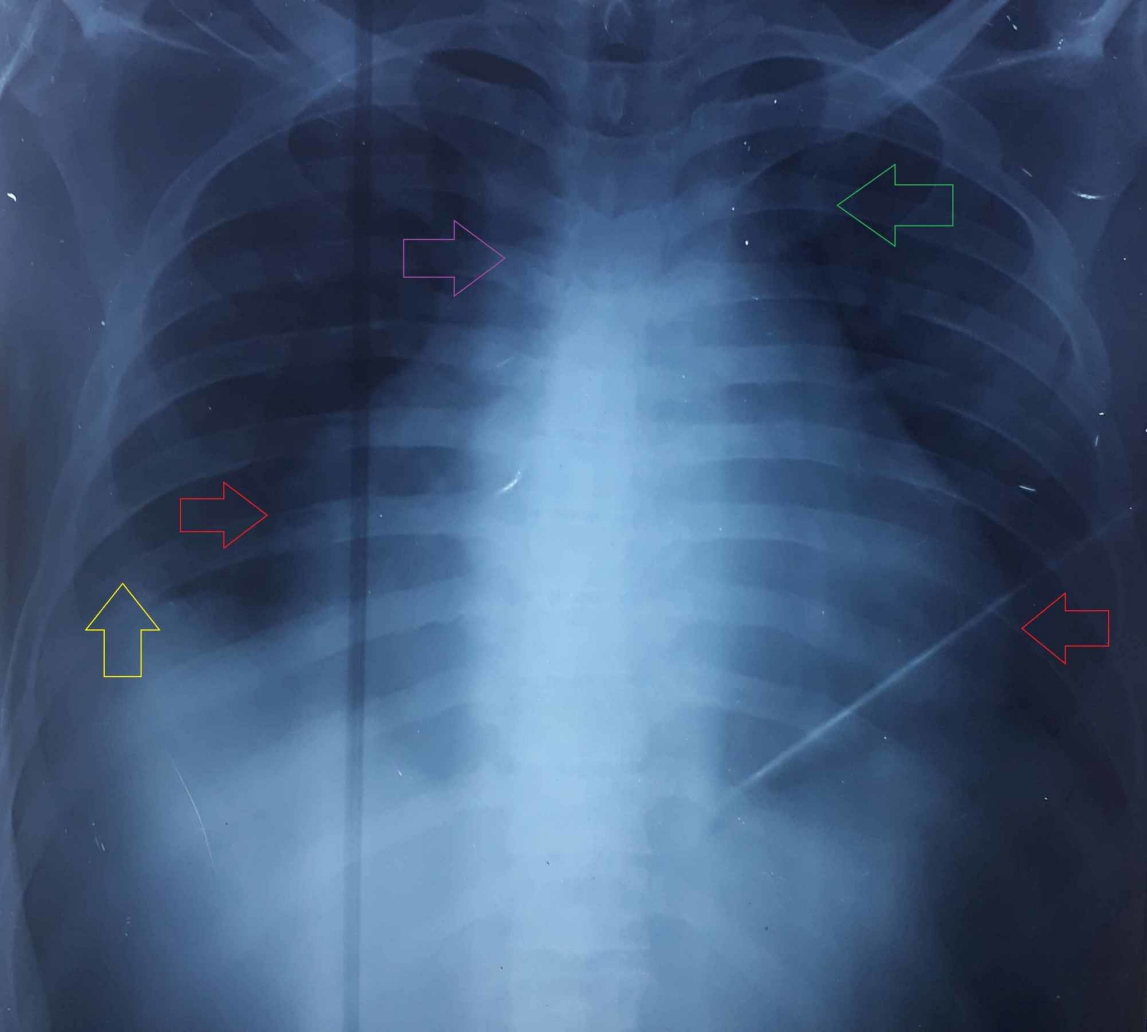
PA chest radiograph showing enlargement of the cardiac silhouette (red arrows), bilateral ILD, enlargement of the azygous vein (purple arrow), peribronchial cuffing (green arrow), and blurring of costophrenic angle (yellow arrow). PA, postero-anterior; ILD, interstitial lung disease.

Lab evaluation on admission revealed hemoglobin (Hb) of 12.5 g/dl [Normal (N) = 13.8-17.2], total leukocyte count (TLC) of 17.8 x 109/L (N = 4-11), high C-reactive protein (CRP) value of 119.4 mg/dL (N = 1.0-3.0), and prothrombin time (PT) of 12.4 s (N = 11-13.5). Electrolytes were normal except sodium (Na) which was decreased up to 116 mEq/L (N = 135-145). Total bilirubin was raised up to 5.14 mg/dL (N = 0.3-1), however, albumin and globulin levels were within the normal range. Chest X-ray (CXR) showed diffuse pulmonary edema with prominent DCM (Figure 2). The patient was initially managed with digoxin, ascard, atorvastatin, levofloxacin, augmentin, and loop diuretic.

His echo demonstrated a decreased EF of 15% with severe tricuspid regurgitation (TR) and LVD. In addition to the findings of previous echo report, akinesia was also noticed in the interventricular septum (IVS) and anterior and inferior walls along with moderate pericardial effusion. Abdominal ultrasound (U/S) revealed hepatomegaly measuring 18.4 cm. Inferior vena cava (IVC) and hepatic veins were found dilated in the Doppler U/S of hepatic and portal veins whereas that of lower limb vessels showed internal echoes in the right popliteal vein representing sluggish flow. No evidence of stenosis and deep vein thrombosis (DVT) was observed.

**Figure 3:**
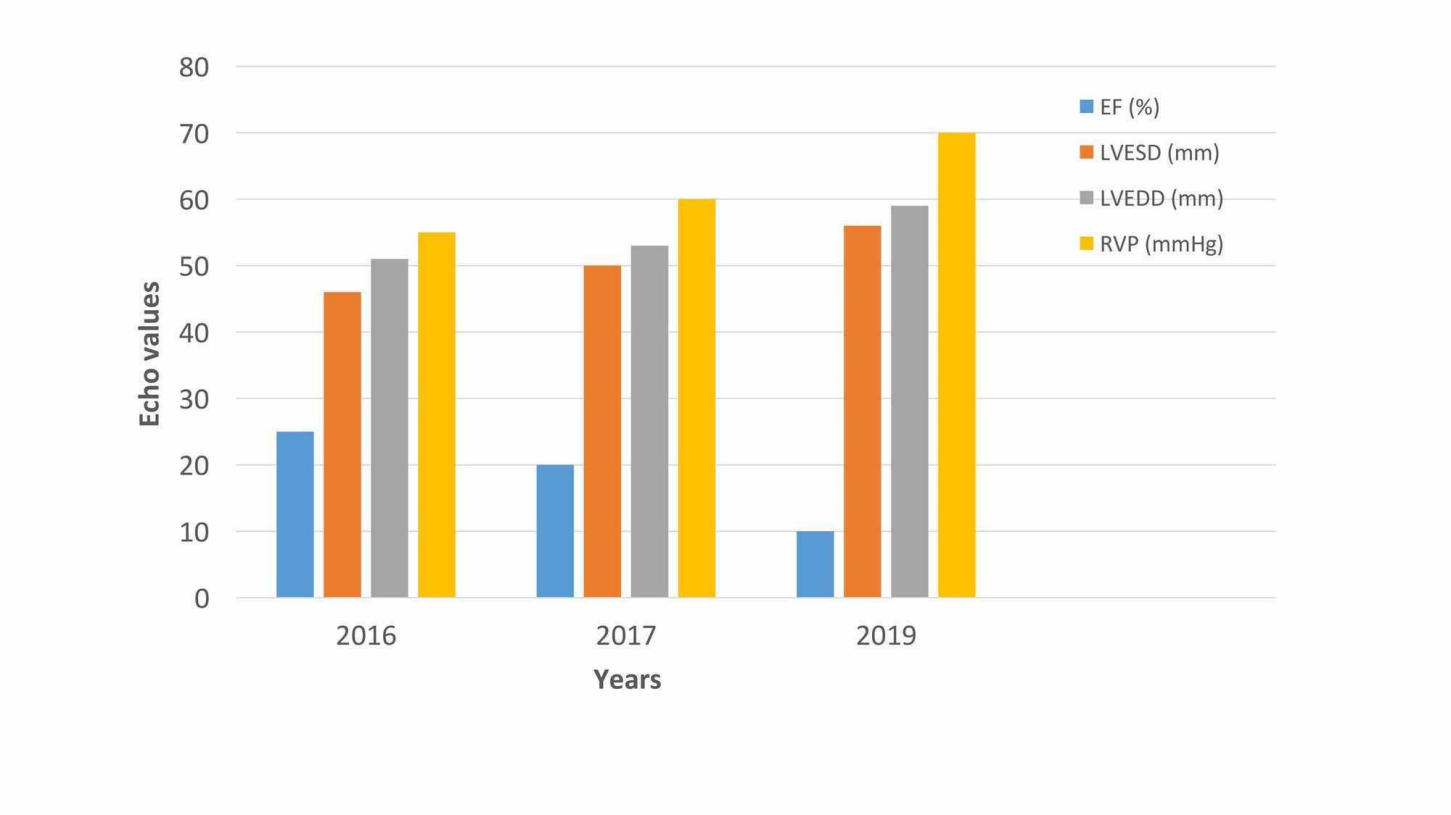
Progressive worsening of echo values since MI in 2016. Echo, echocardiography; MI, myocardial infarction; EF, ejection fraction; LVESD, left ventricular end-systolic diameter; LVEDD, left ventricular end-diastolic diameter; RVP, right ventricular pressure.

Although the patient recovered from cellulitis and pedal edema, there was a progressive declination in his EF which dropped up to 10% along with the worsening DCM (Figure 3).

The BP also dropped up to 60/30 mmHg. Therefore, a worse prognosis of our patient was established and he was discharged with diuretics, beta blockers, nitrates, and anticoagulants. Angiotensin converting enzyme (ACE) inhibitors were avoided due to decreased BP.

## Discussion

Hereditary thrombophilia (also known as hypercoagulability) is an inborn augmented tendency for intravascular thrombi, either venous or arterial, which typically affects young individuals (<45 years) and tends to recur. A significant minority of affected individuals present with a detectable abnormality whereas majority develop thrombosis only when triggered by additional risk factors [6]. Protein C deficiency, a hypercoagulable disorder, is either acquired or congenital in origin; the latter usually transmits to the next generation in an autosomal dominant (AD) fashion. It is said to be heterozygous when the individual has <60% of plasma protein C level, whereas homozygous when there are no detectable levels. The homozygous state usually targets neonates as purpura fulminans neonatalis while the heterozygous state comes to clinical attention usually in the third decade as presented in our case. Heterozygous protein C deficiency manifests as seven-fold increased risk for VTE [7]. Rarely, it can also lead to arterial thrombosis in individuals with a positive family history and/or when triggered by smoking (tobacco), ultimately resulting in premature MI as happened with our patient. Being a major contributor to CVD, premature MI claimed 17.6 million lives globally in 2016 [8]. A study conducted among 337 patients of heterozygous protein C deficiency showed the prevalence of arterial thrombosis in only 7.1% of subjects [9]. A recent work involving 255 consecutive patients, who had endured a STEMI in <35 years of age, revealed protein C deficiency in only one of them (0.4%) [10]. A cohort family study revealed arterial events in only 8% of the 144 subjects with protein C and protein S deficiency [11]. Such a low incidence of correlation among arterial occlusion and protein C and protein S deficiency established the rarity of our case, where a young man presented with acute MI was found to be deficient of these proteins.

Acute coronary syndromes (ACS) may exhibit nonatherosclerotic etiologies, i.e. congenital anomalies of coronary artery, spontaneous coronary artery dissection, vasospasm, use of illicit drug, or hypercoagulable states like our case. The absence of coronary disease must divert the differential towards inherited thrombophilia syndromes, especially in young patients who had suffered from acute MI as in our case. These syndromes mainly include protein C deficiency, protein S deficiency, hyperhomocystenimia, with factor V Leiden deficiency being the most prevalent. Among the aforementioned, our patient only had deficiency of protein C and deficiency of protein S with normal levels of homocysteine and factor V Leiden. It can be hypothesized that increased risk of arterial thrombosis in individuals with protein C deficiency could be attributed to the significant cytoprotective role of protein C pathway [12]. Because protein S binds and assists activated protein C in the degradation of coagulation factors, deficiency of protein S ultimately predisposes to hypercoagulability. Currently, protein S deficiency is found more frequently than protein C deficiency among the general population [13]. Our patient, being deficient of both proteins C and S, had a devastating progress towards DCM due to persistent hypercoagulable state as a result of showing noncompliance to the prescribed medications.

Dilated cardiomyopathy, the most frequent of all cardiomyopathies, is a disorder that involves the myocardium causing ventricular dilatation and impaired systolic function subsequently leading to congestive heart failure (CHF) as ensued in our patient who typically presented with SOB, pleural effusion, hepatic congestion, gross ascites, and pedal edema with a progressively declining EF. There are numerous etiologies for DCM among which IHD (ischemic cardiomyopathy) is the most common. In premature IHD, genetic risk factors dominate environmental factors. Genetic thrombophilia has been found to play a major role in premature atherothrombosis, particularly in young and normolipidemic patients [14] as obvious from our case.

Although associated with an increased risk of thrombosis, some people with protein C and protein S deficiency never develop any complication. On the other hand, symptomatic individuals with VTE or rarely arterial thrombosis are treated using anticoagulants and lifestyle modifications with a good outcome but symptoms may recur. After an established diagnosis of congenital protein C and protein S deficiency as the main causative factor for worsening cardiac output, our patient was discharged with life-long anticoagulants. The prognosis of our patient was declared to be worse as his EF failed to improve despite any therapy.

## Conclusions

Young patients presenting with acute MI, with a positive family history and showing no evidence of atherosclerosis, should be evaluated for deficiency of major anticoagulant proteins especially protein C and protein S. And if found, patients should be counseled to take their medications regularly and they must be informed about all the possible complications of showing noncompliance to the treatment. According to our literature search, we did not find significant data relevant to the incidence of arterial thrombosis in combined deficiency of protein C and protein S. Furthermore, insufficient data about the aggravating risk factors, typical clinical presentation, and treatment strategies for symptomatic collateral protein C and protein S deficiency were published. Ample research on this aspect must be carried out for the proper and timely diagnosis of this rare manifestation of combined protein C and protein S deficiency which itself is a very unusual incident.
